# Arabin-pessary or McDonald Cerclage in Cervical Shortening?

**DOI:** 10.1055/s-0043-1776033

**Published:** 2023-12-23

**Authors:** Aytaj Jafarzade, Sveta Aghayeva, Tamer Mungan, Aydan Biri, Osman Ufuk Ekiz

**Affiliations:** 1 Obstetrics and Gynecology Department, Koru Hospital Ankara, Ankara, Turkey.; 2 Statistic Department, Gazi University, Yenimahalle, Ankara, Turkey.

**Keywords:** cerclage, pessary, cervical insufficiency, McDonald cerclage

## Abstract

**Objective** The aim of the present study is to compare the effectiveness of Arabin pessary and McDonald cervical cerclage on preterm delivery.

**Methods** We conducted a retrospective analysis of data from patients who underwent either Arabin pessary or McDonald cerclage between January 1, 2019, and January 1, 2023. A total of 174 patients were included in the study, with 31 undergoing Arabin pessary and 143 receiving cervical cerclage using the McDonald technique in singleton pregnant women with cervical insufficiency, which applied between 14 and 22 gestational weeks. We included singleton pregnant women with normal morphology, and with normal combined test. The primary outcome was the impact of each method on preterm delivery (< 34 gestational weeks).

**Results** The weeks of cervical cerclage or pessary application were compatible with each other ( *p* < 0.680). The pessary group had a statistically significant longer time to delivery compared with the Cerclage group (cerclage group mean 30.8 c 7.1 standard deviation [SD] versus pessary group mean 35.1 ± 4.4 SD; *p* < 0.002). A statistically significant difference was found between the pessary and cerclage groups in terms of delivery at < 34 weeks ( *p* = 0.002). In patients with cervical length between 25 and 15mm and < 15mm, no significant difference was found between the pessary and cerclage groups in terms of delivery week ( *p* < 0.212; *p* < 0.149). Regardless of the technique applied, no statistically significant difference was observed between cervical length and birth < 34 weeks.

**Conclusion** Our study found that pessary use for cervical insufficiency is statistically more effective than cervical cerclage surgery in preventing preterm births < 34 weeks in singleton pregnancy.

## Introduction

Preterm birth is a serious condition that results in high rates of mortality and morbidity 1 and affects ∼ 10% of all pregnancies. 2 Preterm labor requires high health expenditures and to prevent cervical insufficiency, which is one of the causes of preterm labor, cervical length is measured by transvaginal ultrasonography at the end of the 1 ^st^ trimester and the beginning of the 2 ^nd^ trimester. 3 Women with asymptomatic cervical shortening detected by this low-cost method have a high risk of preterm delivery. 4 There is a meta-analysis that advocates its application during routine controls 5 and argues that routine screening is more appropriate only for high-risk patients, since it does not significantly reduce preterm birth rates. 6 Cervical insufficiency is when dilation and slackening of the cervix (≤ 25 mm) occur before 24 weeks gestation without contraction, bleeding, infection, rupture of the membranes, or labor and history one or more second trimester losses, or prior spontaneous preterm birth at < 34 gestational weeks. 7

Treatment options for patients with cervical shortening include cervical cerclage (abdominal and vaginal), pessary, and observation/medical management. Vaginal cerclage can be performed using either the Shirodkar or the McDonald technique. 8 The pessary applied in cases of cervical insufficiency prevents the cervical shortening and dilation by changing the angle between the uterine lower segment and the cervix. 9 It is minimally invasive, easily accessible, can be applied in an ambulatory setting, and is relatively low-cost, although it may have some negative effects on pregnancy. 2 Studies have shown that the pessary changes the uterocervical angle and reduces cervical dilation and effacement. 10

The main aim of our study is to compare the rate of preterm birth (< 34 weeks of gestation) between pessary and McDonald cerclage methods in patients diagnosed with cervical insufficiency.

## Methods

Our study was conducted retrospectively at the Koru Ankara Hospital between January 1, 2019, and January 1, 2023. We included singleton pregnant women with cervical insufficiency (diagnosed via transvaginal sagittal B-mode ultrasound), normal morphology, and who had undergone the combined test. Patients with uterine malformation, stillbirths, fetal anomalies, preeclampsia, intrauterine grow restriction, placenta previa, and placenta accreta spectrum were excluded from the study.

### Diagnostic Methods for cervical insufficiency (CI)

- **Ultrasound-based diagnosis:** Used when there have been ≥ 1 pregnancy losses or preterm births of 14 to 36 weeks in the past and a cervical length (CL) < 25 mm is measured by transvaginal ultrasound (TVU) before 24 weeks of gestation.
- **Physical examination-based diagnosis:** Used when painless cervical dilatation or prolapsed fetal membranes have been detected on manual or speculum examination before 24 weeks of gestation regardless of whether a history of midtrimester pregnancy loss or preterm birth exists.
- **History-based diagnosis:** Used when there was painless cervical dilatation, leading to recurrent miscarriages in the second trimester and preterm births without other reasons.

Ultrasonography and physical examination were performed simultaneously for those with cervical insufficiency diagnosis. We included patients who underwent pessary or McDonald cerclage due to cervical insufficiency in the study and compared their results. We defined births < 34 weeks as births primary results. Secondary outcomes were the effect of progesterone use on the week of birth, delivery week (< 24, < 28, and < 37), and birthweight.

In patients without vaginal infection, the pessary was placed in the examination room in accordance to the position of the cervical neck, without the need for anesthesia. Following the procedure, the patient was observed for several hours to ensure that there was no discomfort, bleeding, or uterine activity. A speculum inspection was performed to ensure that the pessary was fitted correctly. The pessary was removed in cases of vaginal bleeding, pain and soreness, or rupture of membranes.

All patients underwent cervical cerclage using the McDonald technique while under analgesia. The cervical neck was held with ovarian forceps, carefully pushed in, and sutured at 12, 9, 6, and 3 o'clock with a 5-mm Mersilene tape. Patients who experienced rupture of the amniotic membrane within 48 hours following the procedure were excluded from the study.

We performed statistical analysis using IBM SPSS Statistics for Windows, version 22.0 (IBM Corp., Armonk, NY, USA). Continuous variables were expressed as mean, median, and standard deviation (SD) and were compared between groups using the Mann-Whitney U test. Categorical variables were expressed as the number of patients and percentage and were compared between groups using the Pearson chi-squared test for independent attributes or the Fisher exact test if appropriate. A *p* -value < 0.05 was considered statistically significant.

The present study was conducted in accordance with the principles of the Helsinki Declaration. All subjects participated voluntarily. The participants provided their written informed consent to participate in the present study. The Declaration of Helsinki was adequately addressed, and the study was approved by the Ethics committee of Gazi University.

## Results

A total of 174 patients who underwent cerclage with 31 pessary and 143 McDonald technique due to cervical insufficiency were included in the study. In the study, pessary and cerclage application was performed according to the history in 37 patients (21.3%) and based on physical examination plus ultrasound in 137 patients (78.7%). The diagnosis of cervical insufficiency was made according to the history in 15 patients (48.3%) in the pessary group and as a result of physical examination (physical examination and ultrasound were performed simultaneously) in 16 patients (42.7%). In the cerclage group, 22 patients (15.3%) were diagnosed with cervical insufficiency based on their history and 121 patients (84.7%) on physical examination. No statistically significant difference was observed between the two groups in terms of patient age, body mass index (BMI), parity number, and number of abortions ( Table 1 ).

**Table 1:** Comparison of pessary and cerclage

	Cerclage	Pessary	
	mean	mean	*p-value*
Number of patients ( *n* )	143	31	—
Age, years old (mean ± SD)	30.5 ± 4.4	29.9 ± 3.9	0.526
BMI (mean ± SD)	23.8 ± 3.5	23.5 ± 2.2	0.680
Parity (mean ± SD)	0.45 ± 0.8	0.38 ± 0.5	0.651
Number of abortions (mean ± SD)	0.53 ± 1.0	0.64 ± 1.1	0.611

Abbreviation: BMI, body mass index.

While the cervical effacement was significantly lower in the cerclage group ( *p* = 0.000), there was no significant difference in cervical dilatation between the 2 groups ( *p* = 0.823). The application weeks of cerclage or pessary were compatible with each other ( *p* < 0.680). However, there was a statistically significant difference between the birth weeks of the 2 groups ( *p* < 0.002). The birth week of the pessary group was advanced. The use of progesterone was also statistically significant in the pessary group ( *p* < 0.000). Nevertheless, there was no significant difference in prenatal mortality rates between the 2 groups ( *p* < 0.030) ( Table 2 ).

**Table 2:** Comparison of pessary and cerclage

	Cerclage	Pessary	*p-value*
	Mean	Mean	
Cervical effacement (cm)	12.9 ± 3.7	18.6 ± 5.2	0.000
Cervical dilatation (cm)	26.4 ± 6.4	26.7 ± 5	0.823
Processing week	18 ± 3.1	17.8 ± 2.4	0.680
Birth week	30.8 ± 7.1	35.1 ± 4.4	0.002
Progesterone user	0.8 ± 0.4	1 ± 0	0.000
Prenatal death	0.24 ± 0.4	0.06 ± 0.2	0.030
Birth weight, gr (mean)	1855.85 ± 98.668	2566.48 ± 156.581	0.002

Birthweights of the babies in the pessary group were statistically significantly higher than those in the cerclage group ( *p* = 0.002). In the subgroup analysis, the rate of infants <1,500 g was 16% ( *n* = 5) in the pessary group and 42.6% ( *n* = 61) in the cerclage group. The birth rate of 1,500 to 2,500 gr infants was 9. 6% ( *n* = 3) in the pessary group and 15.3% ( *n* = 22) in the cerclage group. Babies with a birthweight > 2,500 gr comprised 74.3% ( *n* = 23) in the pessary group and 41% ( *n* = 61) in the cerclage group. The number of patients with a birthweight < 1,500 gr and > 2,500 gr in the cerclage group was statistically significantly higher than in the pessary group ( Table 3 ).

**Table 3:** Comparison of the Pessary and Serklyaj groups according to the birth weight of the patients

	Cerclage ( *n* )	Pessary ( *n* )	*p-value*
< 1,500 gr	61	5	0.005 c
1,501 to 2,500gr	22	3	0.411 c
> 2,501gr	60	23	0.001 c

c Chi-squared Test.

When we compared the results of patients using progesterone and not using progesterone between the groups in which cervical cerclage and pessary were applied, and compared the pessary and cerclage groups using progesterone, the gestational week of the pessary group was significantly advanced. However, no significant difference was observed between the cerclage group using and not using progesterone ( Table 4 ).

**Table 4:** Birth weeks of progesterone user and non-user patients among patients who underwent cerclage and pessary

	Cerclage	Pessary	
	Mean	Mean	*p-value*
Progesterone user (Birth week)	30.8 SD ± 7.1	35.1 ± 4.5	0.0021
Progesterone nonuser (Birth week)	30.9 ± 7.3	0	—

When we examined the cervical length of the patients who underwent the procedure as 25–15 mm and < 15mm, the delivery week of the patients with a cervical length of < 15 mm and who had pessary applied on was significantly advanced compared with the other groups ( Table 5 ). There was no statistically significant difference between the week of birth of patients with a cervical length of 25 to 15 mm based on cervical length and who underwent cerclage or pessary ( *p* < 0.212). The week of delivery was not statistically significant in patients with cervical length <15 mm and pessary applied ( *p* < 0.149). There was no statistically significant difference between cervical length and babies born < 34 weeks, regardless of the technique applied ( Table 5 ).

**Table 5:** Birth week by cervical length

	CERCLAGE			PESSARY			
Cervical length (mm)	*n*	Birth week (mean)	Rate birth < 34 w (%)	*n*	Birth week (mean)	Rate birth < 34 w (%)	*p-value*
I 25-15 mm	32	30.5225	( *n* = 12) 37.5%	23	34.3750	( *n* = 5) 21.7%	0.212 c
II < 15mm	111	32.0000	( *n* = 57) 51.4%	8	35.3913	( *n* = 2) 25%	0.149 c
Total	143			31			

c Chi-squared Test.

When we compared the two groups based on the week of birth, rate of birth < 24 weeks of gestation was statistically higher in the cerclage group, and the rate of births > 37 weeks of gestation was statistically higher in the pessary group. There was no statistically significant difference between the other subgroups ( Table 6 ).

**Table 6:** Comparison of groups based on birth weeks in both groups

	CERCLAGE		PESSARY		
Birth week	*n*	Rate (%)	*n*	Rate (%)	*p-value*
< 24	34	23.8	2	6.5	0.031 c
24 to 27 + 6	16	11.2	1	3.2	0.175
28 to 33 + 6	19	13.3	4	12.9	0.954
34 to 36 + 6	24	16.7	3	9.7	0.321
> 37	50	35	21	67.7	0.007
Total	143	100	31	100	

c Chi-squared Test.

**Fig. 1:**
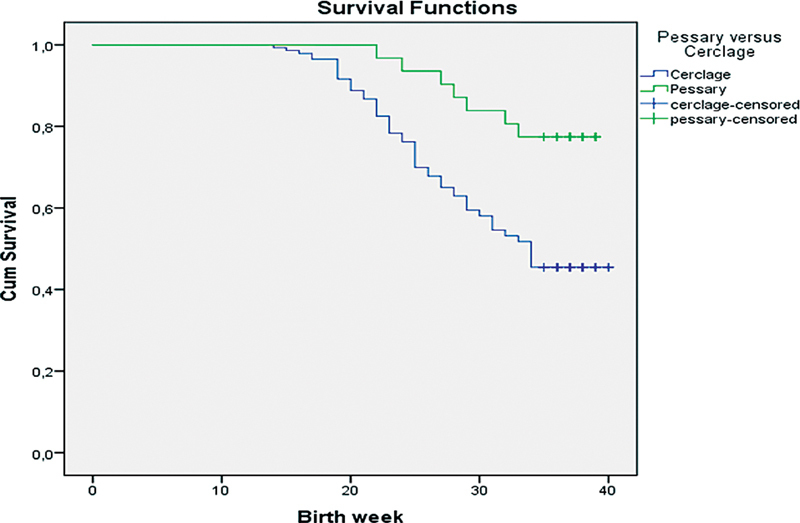
Comparison of Kaplan-Meier survival analysis for pessary and cerclage groups to evaluate the occurrence of preterm birth before 34 weeks in singleton gestation (Breslow-Wilcoxon *p* = 0.02).

However, Kaplan-Meier analysis of survival for the pessary and cerclage groups showed less preterm births before 34 weeks in a singleton pregnancy in the pessary group. The Breslow-Wilcoxon test was used to determine the significant difference between the groups ( *p* = 0.002) ( Fig. 1 ).

## Discussion

In a study conducted by Saccone et al, pregnant women with a cervical length < 25 mm who underwent pessary treatment were compared with those who received observational treatment. The study found that cervical pessary did not reduce the rate of preterm delivery or improve perinatal outcomes. 11

In our study, we compared the results of pessary and cervical cerclage and found that the delivery week of patients who received pessary treatment was significantly more advanced than those who received cervical cerclage ( *p* = 0.002). However, we should consider that the cervical length in the cerclage group was shorter (mean 12.99 ± 3.76 SD for the cerclage group versus mean 18.69 ± 5.23 SD for the pessary group) at the time of pessary and cervical cerclage application. It is important to note that cervical length is known to be the most important factor affecting the week of delivery, when interpreting these results. 12

In their prospective studies, Archarya et al. demonstrated the effectiveness of pessary in both primiparous and multiparous patients with cervical insufficiency. 13

In our study, we found no significant difference in the effectiveness of pessary and cervical cerclage between primiparous and multiparous patients. Specifically, we observed no difference in delivery week between the two groups and found that both groups could benefit from pessary and cervical cerclage application.

While the first randomized controlled trial (PECEP) 14 investigating the efficacy of pessary showed that pessary reduces preterm birth rate and perinatal mortality, studies that did not support these results were also published. 15 Later, the effectiveness of pessary in the prevention of preterm labor was demonstrated in randomized controlled and nonrandomized controlled studies. 16

In our study, some of the patients who underwent cervical cerclage used progesterone while others did not. No statistically significant difference was observed in terms of the week of birth between these two groups, and there was no evidence of progesterone use being beneficial in terms of week of preterm birth. However, as all the patients in the pessary group were using progesterone, a comparison could not be made with the group in which the pessary was applied but progesterone was not used. Nevertheless, when we compared the group that received pessary without progesterone and the group that underwent cervical cerclage without progesterone, we found that the week of delivery was significantly higher in the pessary group.

Melcer et al. conducted a study comparing the results of patients using pessary and progesterone to those using only progesterone due to cervical shortness and found that patients using pessary plus progesterone had a longer gestational week. 17 Although subsequent studies have supported these findings, 18 there is also a study indicating that pessary and progesterone are not more effective than progesterone use alone. 19

The analysis of cervical length in the two groups (Group I: 25 to 15 mm, Group II: < 15 mm) did not reveal any statistically significant difference between the pessary and cerclage groups. Therefore, our findings did not support the hypothesis that cerclage would be more effective in patients with cervical shortness < 15 mm. This is in contrast to the findings of Owen et al., who suggested that cervical cerclage would be more effective when the cervical length was < 15 mm. 20 Nicolaides et al. compared pessary and progesterone treatment in cases with cervical shortness and found that pessary was not superior to progesterone. 21 However, in our study, since progesterone was preferred in patients with cervical length < 15 mm, it is possible that this could potentially reduce any benefit of cervical pessary in this group. Combining methods is not recommended due to lack of benefit according to reviews. 22

In our study, we compared the efficacy of pessary and cerclage in terms of weeks of birth and found birth < 24 weeks of gestation was statistically higher in the Cerclage group, and the rate of births > 37 weeks of gestation was statistically higher in the pessary group. As a primary result, we found a significant difference between the pessary and cerclage groups in terms of delivery at < 34 weeks ( *p* = 0.002). Alfirevic et al. compared vaginal progesterone, pessary, and cerclage outcomes in patients with a history of preterm birth and cervical shortness. 23 The study found that all three methods were not superior to each other in terms of preterm birth and perinatal loss. There was no significant difference in the number of preterm births before 37 weeks between the cerclage, vaginal progesterone, and pessary groups.

Mouzakiti et al. conducted a retrospective study comparing the effectiveness of pessary and McDonald cerclage methods for cervical shortening (< 25 mm) with and without funneling and found that pessary had no superiority over cervical cerclage, but the rate of neonatal intensive care unit (NICU) hospitalization was lower in the pessary group. 24

The limitations of our study are that it is retrospective, with a limited number of cases and does not account for factors that may contribute to cervical insufficiency, such as smoking or ethnicity.

After considering all of this information, we conclude that pessary application appears to be at least as effective as cervical cerclage in preventing preterm labor. Cervical cerclage is a costly procedure that requires general anesthesia and hospitalization, while pessary application is a less expensive procedure which does not require anesthesia or sedation in outpatient conditions and does not require hospitalization afterwards. Therefore, the use of Arabin pessary in appropriate patients appears to be a suitable choice.

## Conclusion

The use of pessary application for cervical insufficiency appears to be equally effective as the surgical procedure of cervical cerclage in preventing preterm delivery. Pessary application may be a preferred option in terms of healthcare expenditures due to its noninvasive, easily accessible, and low-cost nature. Nevertheless, randomized controlled studies are necessary to further investigate the efficacy of pessary application compared with cervical cerclage.
